# Population pharmacokinetic model of high-dose methotrexate in Chinese patients with intracranial germ cell tumors

**DOI:** 10.3389/fphar.2025.1548203

**Published:** 2025-05-02

**Authors:** Jiashu Zhao, Ruoyun Wu, Sitian Zhang, Qian Lu, Ruitao Wang, Yingjun He, Zhigang Zhao, Shenghui Mei

**Affiliations:** ^1^ Department of Pharmacy, Beijing Tiantan Hospital, Capital Medical University, Beijing, China; ^2^ Department of Pharmacy, Beijing Puren Hospital, Beijing, China; ^3^ Department of Clinical Pharmacology, College of Pharmaceutical Sciences, Capital Medical University, Beijing, China; ^4^ School of Basic Medical Sciences, Capital Medical University, Beijing, China; ^5^ Department of Pharmacy, Bijie Maternal and Child Health Hospital, Bijie, China

**Keywords:** methotrexate, intracranial germ cell tumors, population pharmacokinetic model, nonlinear mixed-effects modeling, bleomycin, bilirubin

## Abstract

This study aims to investigate the pharmacokinetics of methotrexate (MTX) in Chinese patients with intracranial germ cell tumors (iGCTs) and to develop a robust population pharmacokinetic (PPK) model. A two-compartment model with an exponential inter-individual variability and a proportional residual model was established using nonlinear mixed-effects modeling. The model was based on 5,470 plasma concentration data points from 505 Chinese iGCT patients, including 370 children. The impact of covariates on model parameters was evaluated using forward addition and backward elimination strategies. Goodness-of-fit plots, bootstrap, visual predictive check and normalized prediction distribution errors were used to assess model performance. In the final model, the clearance of the central compartment (CL) was determined using the following equation $CL=12.88\times {\left(eGFR/102.2\right)}^{0.23}\times {\left(BW/47\right)}^{0.39}\times e^{BLM}\times {\left(TBIL/15.3\right)}^{-0.05}{\times \left(ALB/40.9\right)}^{-0.18}$ (BLM = 0.08 when combined with bleomycin, otherwise = 0). The apparent volume of the central compartment (V_c_) was $V_{c}=72.04\times {\left(\text{BW}/47\right)}^{0.31}$ . The apparent volumes of the peripheral compartments (V_p_) and the inter-compartmental clearance (Q) were fixed as 94.94 L and 1.08 L/h, respectively. Co-administration with bleomycin could increase MTX CL by a factor of 1.08. Elevated total bilirubin and albumin levels were associated with decreased MTX CL. Goodness-of-fit and model evaluation confirmed the final model’s adequacy, stability, and predictive performance. In our study, a PPK model was developed to identify the key factors influencing MTX pharmacokinetics, thereby optimizing and personalizing MTX therapy for Chinese patients with iGCTs.

## 1 Introduction

Intracranial germ cell tumors (iGCTs) are rare neoplasms primarily affecting adolescents, with peak incidence occurring between 12 and 16 years (Kremenevski et al., 2023; Takami et al., 2024). iGCTs demonstrates marked sensitivity to both radiotherapy and chemotherapy, yielding an excellent prognosis, with 5-year overall survival rates ranging from 97% to 100% (Kremenevski et al., 2023).

Methotrexate (MTX), a metabolic-targeting anti-cancer agent, acts by inhibiting both dihydrofolate reductase and thymidylate synthase, thereby blocking purine and pyrimidine synthesis (Shi et al., 2020). High-dose methotrexate (HD-MTX, >500 mg/m^2^) serves as a critical component of chemotherapy for iGCTs treatment (Yamasaki et al., 2020). Although HD-MTX enhances cytotoxicity and anti-tumor efficacy, it is toxic at therapeutic doses, potentially inducing severe bone marrow suppression and multiple organ failure (Mei et al., 2018). To counteract these effects, calcium folinate (CF) rescue is indispensable. Since MTX exhibits substantial intra- and inter-individual variability, plasma concentration monitoring plays a pivotal role in guiding CF dosing and minimizing the side effects (Shi et al., 2020).

Population pharmacokinetic (PPK) models are critical for identifying sources of pharmacokinetic variability, facilitating the optimization of personalized dosing strategies and enhancing therapeutic drug monitoring in specific patient populations (Kiang et al., 2012; Darwich et al., 2021). Published PPK models have indicated that various factors significantly impact MTX pharmacokinetics. Approximately 80%–90% of MTX is excreted via the kidneys, and estimated glomerular filtration rate (eGFR), serum creatinine (SCR), and creatinine clearance (CL_cr_) are key covariates influencing MTX clearance (CL) (RASUVO, 2025; Zhang et al., 2022). Numerous studies have demonstrated that body weight (BW) and body surface area (BSA) are reliable parameters for adjusting MTX dosage, as they correlate strongly with basal metabolic rate and renal function (Shi et al., 2020; Chen et al., 2024; Gao et al., 2021; Panetta et al., 2020). Additionally, several studies have reported the influence of polymorphisms, SLCO1B1 rs2306283 (Schulte et al., 2021), SLCO1B1 rs4149056 (Schulte et al., 2021), ABCC2 rs717620 (Simon et al., 2013), ABCB1 rs1045642 (Kim et al., 2012), ABCG2 rs13120400 (Lui et al., 2018), and MTHFR rs1801133 (Faganel Kotnik et al., 2011) on the CL of MTX. Other factors including age, height, sex, disease type, vertebral body, hematocrit, albumin (ALB), alanine transaminase (ALT), dosage regimens, and co-medications (proton pump inhibitors, penicillin, vancomycin, dexamethasone and nonsteroidal anti-inflammatory drugs) could also influence the pharmacokinetics of MTX (Shi et al., 2020; Zhang et al., 2022; Panetta et al., 2020; Pai et al., 2020; Kawakatsu et al., 2019).

Several PPK models for HD-MTX have been developed in various diseases, including acute lymphoblastic leukemia, osteosarcoma, non-Hodgkin’s lymphoma, primary central nervous system lymphoma, medulloblastoma and malignant brain tumours (Zhang et al., 2022). However, no PPK models have been established in patients with iGCTs. Based on tumor characteristics and patient condition, chemotherapy regimens for iGCTs include CARE (carboplatin, etoposide), ICE (ifosfamide, mesna, cisplatin, etoposide), ACNS0122 (group A: etoposide, carboplatin; group B: ifosfamide, mesna, etoposide), PEI (ifosfamide, mesna, cisplatin, etoposide), EP (etoposide, cisplatin), EC (etoposide, carboplatin), KSPNO G051/G081 (group A: etoposide, carboplatin; group B: ifosfamide, mesna, etoposide) (Nakamura et al., 2021; Frappaz et al., 2021). Our patient received a combination of chemotherapeutic agents, including bleomycin (BLM), vancomycin, etoposide, and cisplatin, all of which may influence the pharmacokinetics of MTX (Kremenevski et al., 2023). This study aims to investigate the pharmacokinetic characteristics of MTX in Chinese patients with iGCTs and develop a robust MTX PPK model for optimizing therapeutic drug monitoring.

## 2 Methods

### 2.1 Patients’ selection and treatment protocol

This study was approved by the Ethics Committees of Beijing Puren Hospital (prll-2024-32) and was performed consistent with the guidelines of the Declaration of Helsinki.

The PPK model for MTX was developed using retrospective data from 505 patients with iGCTs (26.7% adults, 73.3% children) hospitalized at Beijing Puren Hospital between February 2015 and July 2018. Patients included in the analysis data had (i) a confirmed diagnosis of iGCTs; (ii) administration of intravenous MTX dose of ≥0.5 g/m^2^; (iii) therapeutic drug monitoring performed during the treatment course, with at least one MTX concentration measured.

The treatment regimen consisted of MTX and vincristine on day 1, BLM on day 2 and cisplatin on day 3 or 4. MTX (manufactured by Pfizer Australia Pty Ltd., concentration: 1 g in 10 mL) was administered within 24 h of patient admission, with the standard dose calculated based on 1.3 g/m^2^. One-third of the total dose was administered as a bolus intravenous infusion over 1 h, with the remaining dose infused over 11 h. Following discontinuation of MTX for 12 h, CF (13 mg/m^2^) was administered as rescue therapy 5 times, with doses given every 6 h. If required, the CF dose and frequency were adjusted according to the serum MTX concentration.

### 2.2 Data collection

A comprehensive dataset was recorded for the included patients, encompassing: 1) demographic information: sex, age, BW, height, body mass index (BMI), BSA; 2) detailed MTX dosing regimen: date, time, daily dose, frequency, and sampling time; 3) biological parameters: SCR, eGFR, aspartate aminotransferase (AST), ALT, ALB, alkaline phosphatase (ALP), lactate dehydrogenase, total bilirubin (TBIL), total protein; 4) pharmacokinetic data: MTX plasma concentration; 5) concomitant medications: nonsteroidal anti-inflammatory drugs, dexamethasone, BLM, vancomycin, etoposide, and cisplatin. BSA was calculated using the Mosteller formula, while eGFR was determined via the 2008 Schwartz bedside formula for children and the 2021 CKD-EPI formula for adults (Schwartz et al., 2009; Inker et al., 2021).

### 2.3 Plasma concentration of MTX

Fasting venous blood (2 mL) was collected from all patients 24 h after the completion of each MTX infusion, followed by sampling at 12-h intervals up to a maximum of 108 h, without the addition of anticoagulants. The serum MTX concentrations were assessed by enzyme-multiplied immunoassay (Siemens SYVA Viva-E, manufactured by Siemens AG, Germany), with a quantitative range of 0.3–2,600 μmol/L, yet concentrations below 0.3 μmol/L were still detected. The calibrators and quality control samples were analyzed regularly following the manufacturer’s established quality control guidelines.

### 2.4 Statistical analysis

Statistical analyses were performed using SPSS 27.0 (IBM, Armonk, NY, United States). Given that our data does not conform to a normal distribution, all the data is reported as the median (range).

### 2.5 Development of the population pharmacokinetic model

Nonlinear mixed-effects modeling was performed using Phoenix NLME (version 8.3; Certara, St. Louis, MO) with first-order conditional estimation (FOCE-ELS) for parameter estimation and variability. Models were compared based on objective function value (OFV), Akaike information criterion (AIC), and Bayesian information criterion (BIC). Model performance was assessed using visual predictive checks (VPC), bootstrap resampling, and normalized prediction distribution errors (NPDE).

#### 2.5.1 Base model

One-, two-, or three-compartment models were evaluated to identify the optimal structural model. Inter-individual variability (η) in MTX pharmacokinetic parameters was estimated using exponential models (Equation 1). Different types of residual variability (ε), including additive, proportional, and mixed models, were tested to identify the best-fitting model (Equations 2–4). Assumptions were made that the random variables η and ε followed normal distributions with means of 0 and variances of ω^2^ and σ^2^, respectively.

$$\theta =\theta_{TV}\times e^{\eta }$$ (1)

$$C_{obs,i,j}=C_{pred,i,j}+\varepsilon$$ (2)

$$C_{obs,i,j}=C_{pred,i,j}\times \left(1+\varepsilon \right)$$ (3)

$$C_{obs,i,j}=C_{pred,i,j}\times \left(1+\varepsilon_{1}\right)+\varepsilon_{2}$$ (4)

where θ_TV_ denotes the typical population value of the pharmacokinetic parameters, C_obs,i,j_ and C_pred,i,j_ refer to the observed and predicted concentrations, respectively.

#### 2.5.2 Covariate model

To further assess the influence of various covariates on the pharmacokinetic variability of MTX, a stepwise approach, including forward inclusion and backward elimination, was used. Covariates were retained in the final model if the objective function value (OFV) decreased by 6.64 (P < 0.01) for forward inclusion or increased by 10.83 (P < 0.001) for backward elimination. Continuous covariates were standardized to their respective median values before analysis.

#### 2.5.3 Goodness-of-fit and model evaluation

The following goodness-of-fit plots were used to assess the fit between the base and final models: 1) observed concentration (DV) versus population-predicted concentration (PRED); 2) conditional weighted residuals (CWRES) versus PRED; 3) CWRES versus time after dose (TAD); 4) CWRES versus standard normal quantiles. To assess the robustness of the model, a bootstrap analysis (1,000 runs) was performed, with the median parameter estimates compared to those from the final model. Additionally, a Visual Predictive Check (VPC) was conducted, comparing the 5th to 95th percentiles of the simulated prediction intervals with the distribution of observed data. NPDE was obtained from 1,000 Monte Carlo simulations. Graphical diagnostics, including histograms, quantile-quantile plots, and NPDE versus TAD and PRED plots, are presented. Statistical tests, including the Wilcoxon signed-rank test for the mean, Fisher’s test for variance, and the Shapiro-Wilk test for normality, were conducted to evaluate the model. All analyses were conducted using R software (version 4.2.3, https://www.r-project.org).

## 3 Results

### 3.1 Characteristics of enrolled participants

A total of 5470 MTX plasma concentrations were collected from 505 patients (357 females and 148 males) with iGCTs at Beijing Puren Hospital. Patient ages ranged from 3 to 48 years, with children making up 73.3% of the cohort. The median BW was 47 kg and the median eGFR was 102.2 mL/min/1.73 m^2^ (from 41.57 to 446.22 mL/min/1.73 m^2^). Concurrent use of BLM was observed in 58.67% of patients. Demographic characteristics, laboratory results, and concomitant medications are summarized in Table 1.

**TABLE 1:** Characteristics of patients in the population pharmacokinetic model.

Variable	Median (range)
No. of. subjects	505
No. of. concentration sample	5,470
Sex (Male/Female)	357/148
Age (years)	14.00 (3.00–48.00)
Body weight (kg)	47.00 (14.00–121.00)
Height (cm)	155.00 (103.00–194.00)
BMI (kg/m^2^)	19.19 (11.14–36.53)
BSA (m^2^)	1.42 (0.63–2.47)
MTX concentration (μmol/L)	1.2 (0.01–55.80)
Scr (μmol/L)	62.00 (9.00–147.00)
eGFR (mL/min/1.73 m^2^)	102.20 (41.57–446.22)
AST (U/L)	26.00 (8.00–663.00)
ALT (U/L)	17.00 (3.00–530.00)
ALB (g/L)	40.90 (30.60–52.30)
ALP (U/L)	110.00 (20.00–593.00)
LDH (U/L)	212.00 (80.00–1,059.00)
TBIL (μmol/L)	15.30 (3.40–78.50)
TP (g/L)	62.80 (46.90–87.80)
Concomitant medications	
Non-steroidal anti-inflammatory drugs	1,580 (98.50%)
Dexamethasone	751 (46.82%)
Bleomycin	941 (58.67%)
Vancomycin	1,436 (89.53%)
Etoposide	12 (0.75%)
Cisplatin	627 (39.09%)

BMI, body mass index; BSA, body surface area; MTX, methotrexate, Scr serum creatinine; eGFR, estimated glomerular filtration rate; AST, aspartate aminotransferase; ALT, alanine aminotransferase; ALB, albumin; ALP, alkaline phosphatase; LDH, lactate dehydrogenase; TBIL, total bilirubin; TP, total protein.

### 3.2 Development of population pharmacokinetic model

A two-compartment model with first-order elimination best described the pharmacokinetics of MTX, with parameters including CL, central compartment volume (V_c_), peripheral compartment volume (V_p_), and inter-compartmental clearance (Q). Study findings identified eGFR as the most influential factor in MTX CL ΔOFV = −122.058, P < 0.01), with higher eGFR associated with faster CL. Furthermore, BW, BLM, TBIL, and ALB were also significant covariates influencing MTX CL, while BW significantly impacted MTX V_c_. Other covariates did not show statistically significant effects and were excluded from the model. The model development process for the final model is detailed in Table 2.

**TABLE 2:** Results in the model development procedure of final model.

Model no.	Model description	OFV	∆OFV	*P* value
Forward addition				
1	Base model	10,909.702		
2	Add eGFR on CL in model 1	10,787.644	−122.058	<0.01
3	Add BW on CL in model 2	10,665.676	−121.968	<0.01
4	Add BW on Vc in model 3	10,544.391	−121.285	<0.01
5	Add BLM on CL in model 4	10,470.757	−73.634	<0.01
6	Add TBIL on CL in model 5	10,438.016	−32.741	<0.01
7	Add ALB on CL in model 6	10,421.315	−16.701	<0.01
Backward elimination				
8	Remove eGFR on CL from model 7	10,584.303	162.988	<0.001
9	Remove BW on CL from model 7	10,678.173	256.858	<0.001
10	Remove BW on Vc from model 7	10,547.973	126.658	<0.001
11	Remove BLM on CL from model 7	10,500.991	79.676	<0.001
12	Remove TBIL on CL from model 7	10,451.224	29.909	<0.001
13	Remove ALB on CL from model 7	10,438.016	16.701	<0.001

eGFR, estimated glomerular filtration rate; BW, body weight; BLM, bleomycin; TBIL, total bilirubin; ALB, albumin.

Random residual variability was optimally characterized using a proportional error model. The corresponding mathematical equations are provided in Equations.5–8.

$$\text{CL}=12.88\times {\left(\text{eGFR}/102.2\right)}^{0.23}\times {\left(\text{BW}/47\right)}^{0.39}\times e^{\text{BLM}}\times {\left(\text{TBIL}/15.3\right)}^{‐0.05}{\times \left(\text{ALB}/40.9\right)}^{‐0.18}$$

BLM=0.08 when combined with BLM,otherwise=0 (5)

$$V_{c}=72.04\times {\left(\text{BW}/47\right)}^{0.31}$$ (6)

$$Q=1.08\;\left(\text{fixed}\right)$$ (7)

$$V_{p}=94.94\;\left(\text{fixed}\right)$$ (8)

where 12.88 (L/h) and 72.04 (L) are the typical values for CL and V_c_, respectively. Based on the stable estimates from the base model, Q and V_p_ were fixed at 1.08 L/h and 94.94 L, respectively. This was due to limited data, which caused significant shrinkage in the estimation of their interindividual variability, necessitating their fixation at zero (Savic and Karlsson, 2009).Our study was similar to the two-compartment PPK model for both children and adults established by Nader et al. (2017), with typical values of CL, V_c_, Q and V_p_ 15.7 L/h, 79.2 L, 0.97 L/h and 51.4 L, respectively. Except for the typical value of V_p_, which slightly falls outside the range of previous studies (51.4 L vs. 94.94 L), all other parameters were within the ranges of earlier models.

eGFR, SCR, CL_cr_ are the most important covariates affecting MTX CL (RASUVO, 2025; Zhang et al., 2022). Due to the lack of urine data in our study, CL_cr_ could not be accurately estimated in children. Therefore, it was not included as a tested covariate. Compared to SCR (ΔOFV = − 61.22, P < 0.01), eGFR (ΔOFV = −122.058, P < 0.01) was a more significant predictor of MTX CL, with higher eGFR correlating to increased CL, aligning with previous studies (Panetta et al., 2020; Kawakatsu et al., 2019). In the development of our final model, both BW and BSA significantly affected MTX CL, with ∆OFV values of −121.968 and 116.073 (P < 0.01), respectively. Due to collinearity between the two covariates, we explored two different approaches for their inclusion in the final model. In the final model, BW outperformed BSA in -2LL (10,421.315 vs. 10,423.642), AIC (10,443.315 vs. 10,445.642), and BIC (10,515.992 vs. 10,518.319), therefore, BW was selected as the final covariate. Other selected covariates were TBIL, ALB and BLM. Median values of eGFR, BW, TBIL, and ALB were 102.2 mL/min/1.73 m^2^, 47 kg, 15.3 μmoI/L, and 40.9 g/L, respectively. Coefficients between CL and eGFR, BW, TBIL, and ALB were 0.23, 0.39, −0.05, and −0.18, respectively. For V_c_, the coefficient associated BW is 0.31. Additionally, BLM was assigned a value of 0.08 when co-medicated. Detailed information on parameter estimates, relative standard errors, 95% confidence intervals, interindividual variability, residual variability, and bootstrap results are provided in Table 3.

**TABLE 3:** Parameter estimates and bootstrap results of methotrexate population pharmacokinetic model.

Parameter	Base model		Final model		Bootstrap	
	Estimate (%RSE)	95% CI	Estimate (%RSE)	95% CI	Median (%RSE)	95% CI
Vc (L)	72.57 (0.02)	(70.31,74.82)	72.04 (0.01)	(70.00, 74.09)	72.03 (0.02)	(69.65, 74.60)
Vp (L)	94.94 Fixed	—	94.94 Fixed	—	94.94 Fixed	—
CL (L/h)	13.19 (0.02)	(12.77,13.61)	12.88 (0.02)	(12.49, 13.26)	12.85 (0.02)	(12.41, 13.38)
Q (L/h)	1.08 Fixed	—	1.08 Fixed	—	1.08 Fixed	—
eGFR on CL (L/h)	—	—	0.23 (0.14)	(0.17, 0.29)	0.23 (0.13)	(0.17, 0.29)
BW on CL (L/h)	—	—	0.39 (0.09)	(0.32, 0.46)	0.39 (0.09)	(0.32, 0.46)
BW on V (L)	—	—	0.31 (0.12)	(0.24, 0.39)	0.31 (0.12)	(0.24, 0.39)
BLM on CL (L/h)	—	—	0.08 (0.16)	(0.05, 0.10)	0.08 (0.15)	(0.05, 0.10)
TBIL on CL (L/h)	—	—	−0.05 (−0.27)	(−0.07, −0.02)	−0.05 (−0.27)	(−0.07, −0.02)
ALB on CL (L/h)	—	—	−0.18 (−0.40)	(−0.31, −0.04)	−0.18 (−0.38)	(−0.31, −0.04)
IIV Vc(CV%)	1.20	—	0.32	—	—	—
IIV CL(CV%)	5.07	—	2.98	—	—	—
σ (proportional)	0.38 (0.02)	(0.36,0.39)	0.37 (0.02)	(0.36, 0.38)	0.37 (0.02)	(0.36, 0.38)

RSE, residual Standard Error; CI, confidence interval; IIV, inter-individual variability, CV% coefficient of variation, CL, clearance of the central compartment, V_c_ apparent volume of the central compartment, V_p_ apparent volumes of the peripheral compartments, Q inter-compartmental clearance, eGFR, estimated glomerular filtration rate; BW, body weight; BLM, bleomycin; TBIL, total bilirubin; ALB, albumin.

### 3.3 Goodness-of-fit and model evaluation

**FIGURE 1:**
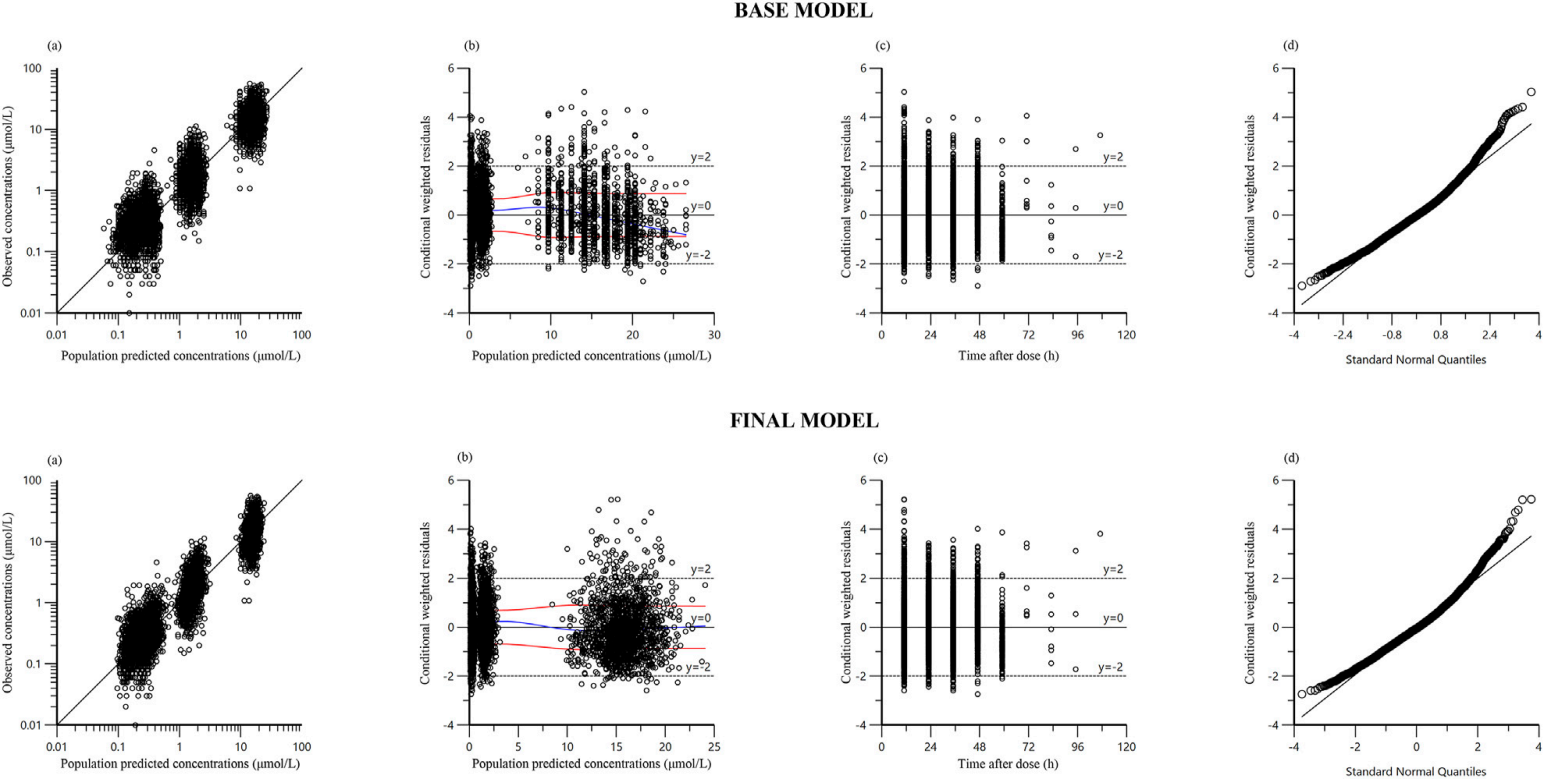
Diagnostic goodness‐of‐fit plots of base model and final model: **(a)** observed versus population predicted concentration (PRED): Data points represent the observed concentrations, with the line indicating perfect prediction. Points should be close to the line, reflecting the accuracy of the model’s predictions; **(b)** conditional weighted residual (CWRES) versus PRED: The residuals should be randomly distributed around zero without any clear trends, indicating no systematic errors in the model; **(c)** CWRES versus time after dose (TAD): Residuals should be evenly distributed across all time points, suggesting that the model is not biased over time; **(d)** quantile–quantile plots of CWRES: Points should closely align with the line, demonstrating that the residuals follow a normal distribution.

Figure 1 presents the goodness-of-fit plots for both the base and final models. Scatter plots of observed concentrations vs. PRED (Figure 1a) demonstrate strong correlations, underscoring good predictive performance. CWRES vs. PRED and CWRES vs. TAD plots (Figures 1b,c) indicate residuals within ±2 standard deviations, evenly distributed around zero, affirming model reliability. The quantile-quantile (Q-Q) plots (Figure 1d) demonstrate that the random effects in the final model are normally distributed.

**FIGURE 2:**
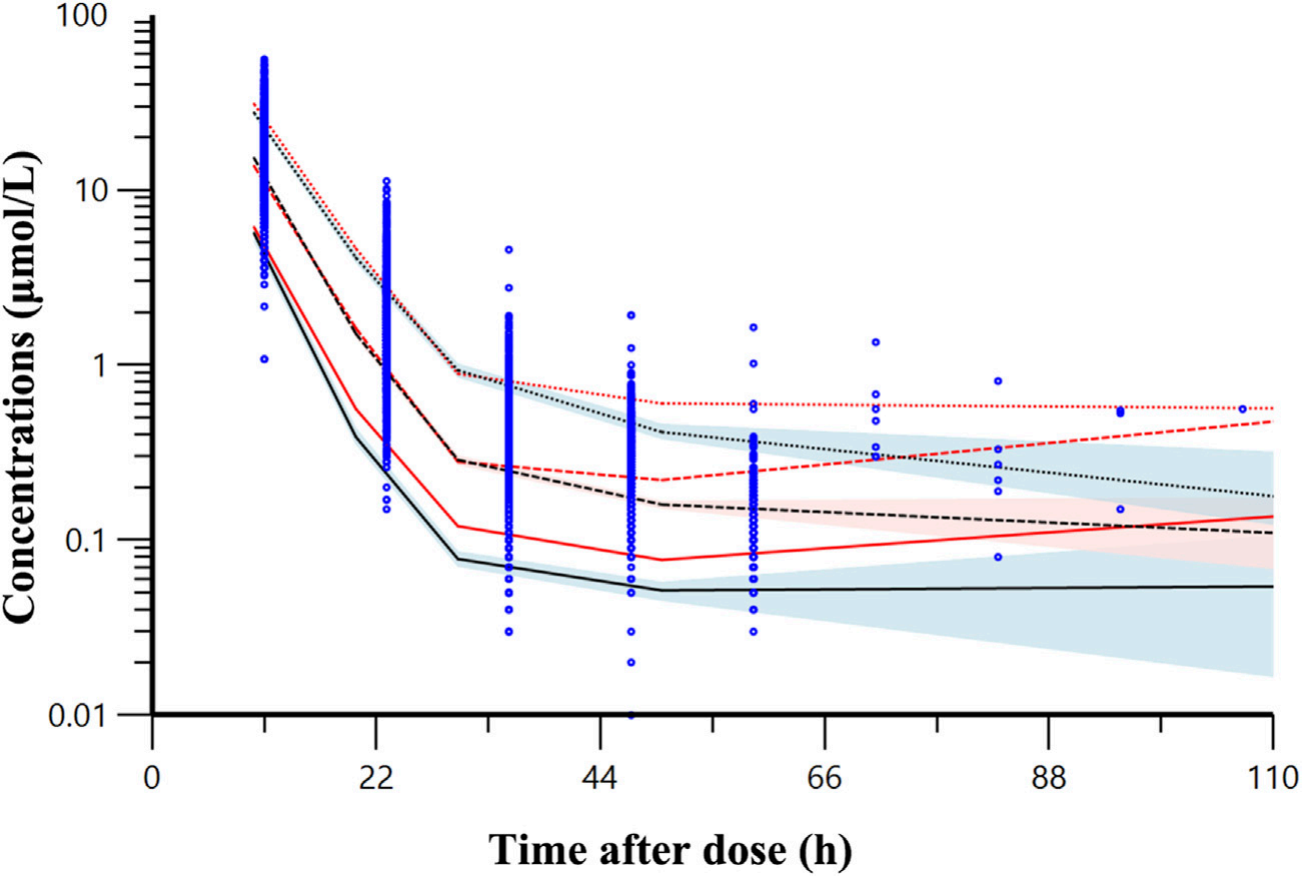
Visual predictive check result of final model. The observed data for methotrexate are denoted by blue dots. The red lines represent observed quantiles while the black lines show predicted quantiles (solid for fifth, dashed for 50th, and dotted for 95th percentiles). The red and blue regions respectively represent the 95% confidence intervals for the fifth, 50th and 95th percentile of the predicted concentrations.

A bootstrap analysis comprising 1,000 runs was conducted for the final model. Parameter estimates from the base model, final model, and bootstrap analysis (Table 3) exhibited close agreement, reflecting consistency. The VPC (Figure 2) illustrates that most observed concentrations fall within the 90% prediction intervals, validating acceptable model performance.

**FIGURE 3:**
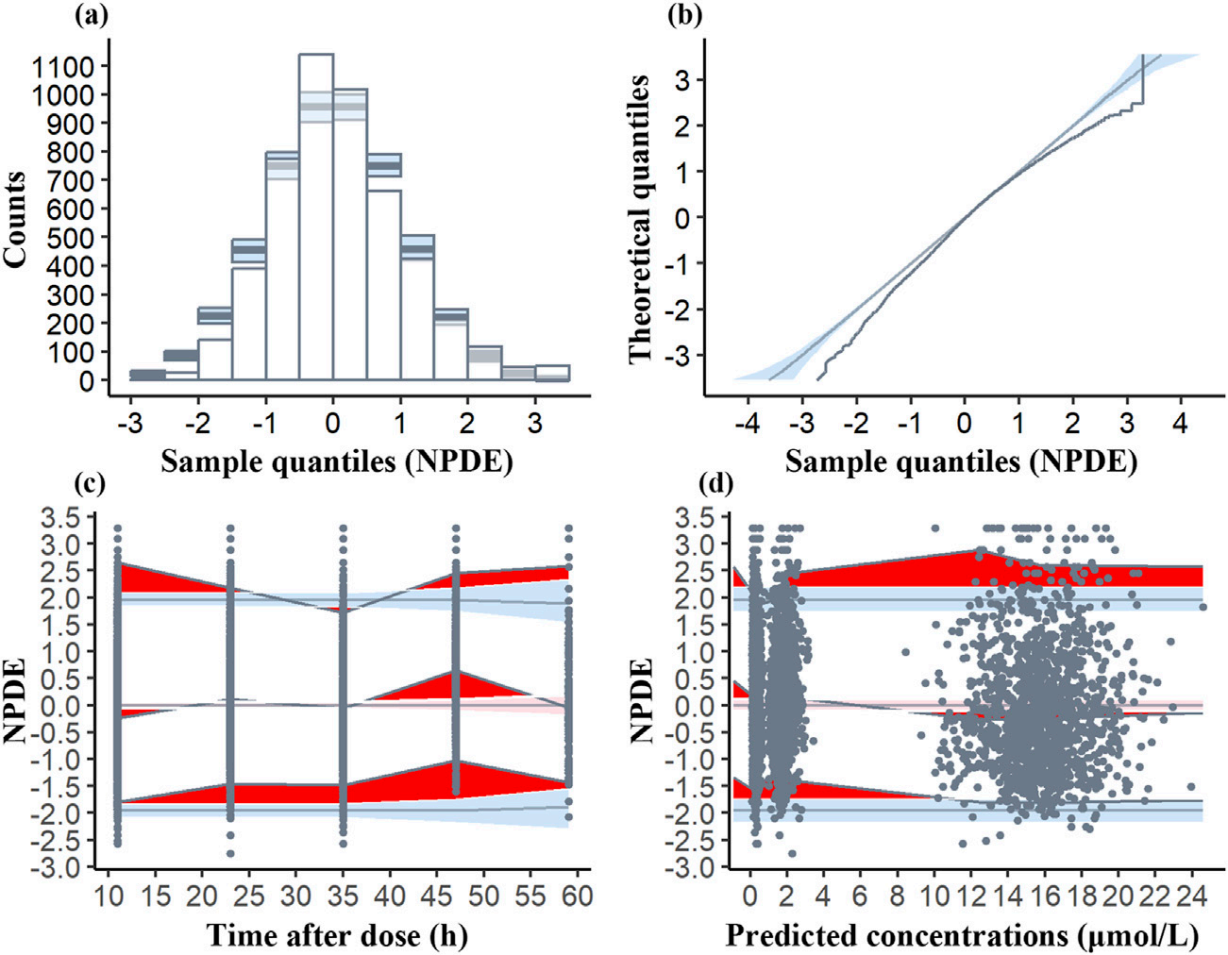
Normalized prediction distribution error (NPDE) plots of the model. **(a)** Histogram of NPDE distribution compared to the theoretical distribution with the blue shaded area indicating the expected range of NPDE values if the model is correctly specified. **(b)** Q-Q plot of NPDE vs. theoretical distribution with the blue fields representing the expected distribution under the null hypothesis. **(c)** NPDE vs. time after dose. The deep red area shows NPDE values outside the expected range, the light red area is the 95% CI for the median NPDE, and the light blue areas are the 95% CI for the 5th and 95th percentiles. Black dots are the observed NPDE values. **(d)** NPDE vs. predicted concentrations, with similar graphical elements as in **(c)**.

The mean NPDE (0.1072) deviates from zero, and the variance (0.9521) is slightly below one. The t-test (p < 0.001) reveals significant deviation, whereas the Fisher variance test (p = 0.0453) suggests variability concerns. However, the histogram (Figure 3) is nearly symmetric, and the Q-Q plot demonstrates that most NPDE values align with the normal distribution, exhibiting only minor tail deviations. These findings support the model’s robustness.

## 4 Discussion

Our study presents the first MTX PPK model in iGCTs patients, based on a large cohort of 505 subjects and 5,470 concentration samples. A two-compartment model with first-order elimination was established.

Approximately 80%–90% of MTX is excreted through the kidneys, with higher eGFR correlating to increased CL. eGFR utilizes specific formulas that normalize values to a standard body surface area of 1.73 m^2^, allowing for accurate comparison of kidney function across various ages and body sizes (Kawakatsu et al., 2019). Besides, Barreto et al. proposed that cystatin C-based eGFR equations may provide a more accurate estimate of MTX CL (Barreto et al., 2022). Due to the absence of cystatin C data in our dataset, the related eGFR calculation was not performed.

In the combined medication covariate analysis, only BLM was found to be significant. Cisplatin has significant nephrotoxicity, and MTX is primarily excreted through the kidneys (Tang et al., 2023). However, cisplatin was excluded as a final covariate, possibly because it is administered 2–3 days after MTX, by which time a large portion of MTX has already been eliminated and many patients lacked MTX concentrations (with records available for 53.07% at 48 h, 26.53% at 60 h, and only 1.39% at 72 h), limiting the analysis of its effects. Similar to our results, Colom et al. (2009) observed no association between cisplatin and renal impairment and excluded this covariate from the final model.

MTX CL increases by 1.08-fold when co-administered with BLM. Concurrent use of BLM with nephrotoxic drugs, such as MTX and cisplatin, can cause renal dysfunction (Dalgleish et al., 1984). Approximately 65% of BLM is eliminated via kidneys, renal impairment can reduce its CL, leading to drug accumulation and an increased risk of pulmonary toxicity (Dalgleish et al., 1984; BLENOXANE, 2010; Sleijfer et al., 1996). However, the influence of BLM on MTX remains unexplored. Our study found that BLM slightly accelerates the excretion of MTX, and the underlying mechanism of this finding requires further investigation. The potential mechanisms may be similar to those of other drugs that accelerate MTX CL, including activation of metabolic enzymes (Song et al., 2021), upregulation of transporters to enhance excretion (El Masri et al., 2023), modulation of oxidative stress and the glutathione system to reduce toxic metabolites (Mahmoud et al., 2019), and activation of detoxifying enzymes to speed up drug inactivation (Widemann et al., 2004).

BW is associated with variations in fat, lean tissue, and water content across individuals (Cheymol, 2000). Given that the majority of our cohort consisted of children (73.3%), BW is strongly correlated with renal function development. The results of this study indicated that an increase in BW correlated with higher V_c_ and CL, consistent with findings from previous models (Shi et al., 2020; Schulte et al., 2021; Gallais et al., 2021; Medellin-Garibay et al., 2020).

Our study identified that increased TBIL was associated with decreased MTX CL. Nakano et al. found that elevated TBIL is an independent risk factor for delayed MTX elimination, which is consistent with our findings (Nakano et al., 2021). Biliary excretion accounts for 10% or less of the administered dose of MTX (Hospira and Methotrexate injection, 2011), which may contribute to delayed MTX elimination in patients undergoing HD-MTX therapy with severe bilirubin excretion dysfunction.

Previous studies have tested ALB as a covariate but found no impact on MTX CL (Panetta et al., 2020; Kawakatsu et al., 2019; Gallais et al., 2021; Zang et al., 2019). Mao et al. (2022) and Pai et al. (2020) observed that decreased ALB resulted in lower MTX CL. They explained that low ALB reduces oncotic pressure, leading to increased third-space fluid, where MTX is distributed and retained, thereby delaying its elimination (Kataoka et al., 2021). Regarding clinical outcomes, Amitai et al. (2020) found that lower ALB levels were an independent risk factor for HD-MTX associated acute kidney injury in patients with hematological malignancies whereas Li et al. (2019) identified higher ALB as a risk factor for high-dose MTX-induced hematotoxicity in children with acute lymphoblastic leukemia. Additionally, Cai et al. (2016) found that lower ALB was independent predictive factors for CNS relapses in diffuse large B-cell lymphoma patients, who received intrathecal chemotherapy (MTX plus cytarabine) if considered at high risk for relapse. Our study revealed that the decreased ALB resulted in higher CL of MTX. This can be explained by the fact that MTX is about 50% protein-bound, and lower ALB levels may increase the unbound fraction of MTX, thereby enhancing its clearance and metabolism (Lukare Medical, 2022). The increase in third-space fluid and the decrease in MTX protein binding were two counterbalancing effects on MTX clearance. For lymphoma patients (previous studies conducted in this population), pleural effusions are relatively common, occurring in 20%–30% of cases (Vakil et al., 2018; Weick et al., 1973). Therefore, the increase in third-space fluid may outweigh the effects of decreased MTX protein binding on MTX CL. For our patients diagnosed with iGCTs, pleural effusions were less common. Therefore, the reduction in MTX protein binding was the predominant factor.

Consistent with previous studies (Shi et al., 2020; Mei et al., 2018; Simon et al., 2013; Gallais et al., 2021; Medellin-Garibay et al., 2020; Zang et al., 2019; Zhang et al., 2015; Faltaos et al., 2006; Aumente et al., 2006), we did not observe the effect of ALT, AST and ALP on PPK parameters of MTX. Only two studies by Kawakatsu et al. (2019) and Dupuis et al. (2008) identified ALT as an independent predictor of CL, Vc and Vp of MTX. They stated that liver dysfunction may hinder the metabolic process, resulting in delayed CL of MTX (Dupuis et al., 2008). Moreover, elevated ALT can impair ALB production, potentially affecting MTX distribution and metabolism (Kawakatsu et al., 2019). As for comedications, non-steroidal anti-inflammatory drugs may reduce MTX CL by inhibiting renal prostaglandin synthesis, competing with protein binding, and interfering with organic anion-mediated renal excretion (Joerger et al., 2006). Proton pump inhibitors and penicillin can decrease MTX renal secretion by inhibiting hydrogen ion elimination (Joerger et al., 2006). Dexamethasone likely increased MTX CL due to enhanced liver metabolism of MTX (Shi et al., 2020; Panetta et al., 2020). Vancomycin could also increase MTX CL, although the exact mechanism remains unclear (Panetta et al., 2020). However, our study failed to find the influence of these covariates on model parameters during model development, consistent with other studies (Shi et al., 2020; Mei et al., 2018; Kim et al., 2012; Medellin-Garibay et al., 2020).

This study has several limitations: (1) Limited sample size during the distribution phase may introduce bias in estimating the volume of distribution. (2) Incomplete MTX concentration data limited the analysis of the interactions with co-administered medications. (3) The absence of urine MTX concentration data hindered accurate estimation of CL_cr_ in children. (4) The lack of genetic information precluded the evaluation of genetic factors. (5) iGCTs are rare neoplasms that account for approximately 3%–5% of all primary CNS tumors in children (Yeo et al., 2023), therefore we could not get enough samples for external validation. Future studies should integrate these data to refine MTX pharmacokinetic modeling and conduct external validation to enhance the generalizability of the findings.

## 5 Conclusion

A robust MTX PPK model for Chinese patients with iGCTs was successfully developed. In the final model, MTX CL was positively correlated with higher eGFR, increased BW, co-administration of BLM, and lower level of ALB and TBIL, while MTX V_c_ increased with elevated BW. This model demonstrated strong stability and reliable predictive performance, offering potential for optimizing individualized MTX therapy in iGCT patients and contributing to more personalized treatment strategies.

## Data Availability

The raw data supporting the conclusions of this article will be made available by the authors, without undue reservation.
